# Does music support executive functions and affective responses during acute exercise? A systematic review and meta-analysis

**DOI:** 10.3389/fpsyg.2025.1714707

**Published:** 2026-01-08

**Authors:** Andrew Danso, Julia Vigl, Friederike Koehler, Keegan Knittle, Joshua S. Bamford, Patti Nijhuis, Eero A. Haapala, Ming Yu Claudia Wong, Shannon E. Wright, Margarida Baltazar, Nora Serres, Niels Chr. Hansen, Andrea Schiavio, Suvi Saarikallio, Geoff Luck

**Affiliations:** 1 Centre of Excellence in Music, Mind, Body and Brain, Jyväskylä, Finland and Department of Music, Arts and Culture Studies, University of Jyväskylä, Jyväskylä, Finland; 2 Department of Psychology, University of Innsbruck, Innsbruck, Austria; 3 Faculty of Sport and Health Sciences, University of Jyväskylä, Jyväskylä, Finland; 4 Centre for the Study of Social Cohesion, University of Oxford School of Anthropology and Museum Ethnography, Oxford, United Kingdom; 5 Institute of Biomedicine, School of Medicine, University of Eastern Finland, Kuopio, Finland; 6 Children’s Health and Exercise Research Centre, Faculty of Health and Life Sciences, University of Exeter, Exeter, United Kingdom; 7 The Education University of Hong Kong, New Territories, Hong Kong, China; 8 Department of Psychology, University of the Fraser Valley, Abbotsford, BC, Canada; 9 Department of Psychology and RITMO Centre for Interdisciplinary Studies in Rhythm, Time and Motion, University of Oslo, Oslo, Norway; 10 Cognitive Musicology and Performance Science Lab, Department of Communication and Psychology, Aalborg University, Aalborg, Denmark; 11 Centre for Music Education and Human Flourishing, School of Arts and Creative Technologies, University of York, York, United Kingdom

**Keywords:** affect, attention, cognition, executive function, exercise, inhibitory control, music, sport

## Abstract

**Introduction:**

Maintaining a steady running pace despite physical or mental fatigue often engages executive functions. These functions may contribute to sustaining exercise participation by regulating cognitive and affective responses to the demands of physical exercise. Research on both music and acute exercise independently shows engagement of executive functions and affective responses, with exercise intensities influencing outcomes. However, the combined effects of music and acute exercise on executive functions and affective outcomes remain underexplored.

**Methods:**

Accordingly, this review examines how music may interact with executive functions and affective responses during acute exercise.

**Results:**

Ten studies met the inclusion criteria, with nine providing data for effect size calculations across 21 intervention arms. Narrative synthesis indicated context-dependent patterns between music and acute exercise combinations, particularly at low-to-moderate exercise intensities. Meta-analyses report non-significant effects of music and acute exercise on attention allocation, inhibitory control, and core affect. A meta-regression pooling 18 effect sizes from nine studies suggested that higher exercise intensities and older mean participant age were associated with smaller effects of music and explained a substantial proportion of between-study variance, although residual heterogeneity remained high and these findings should be interpreted cautiously. A descriptive subgroup analysis showed a decreasing pattern across exercise intensities (low: *g* = 3.99; moderate: *g* = 0.99; high: *g* = 0.28), though substantial heterogeneity persisted, and the reported effects do not appear to generalize consistently across studies.

**Discussion:**

The current synthesised evidence appears inconclusive regarding music’s influence on executive functions and affective responses during acute exercise.

**Systematic review registration:**

https://www.crd.york.ac.uk/PROSPERO/view/CRD42023465958.

## 1 Introduction

Maintaining a steady running pace despite physical or mental fatigue often engages executive functions (Diamond, 2013; Audiffren and André, 2019). Executive functions constitute a set of top-down cognitive processes (Diamond, 2013; Miyake et al., 2000). They are engaged when automatic responses or reliance on instinct and intuition are inadequate, inappropriate, or insufficient to meet task demands (Diamond, 2013). According to Miyake et al. (2000) and Diamond (2013), executive functions span three core components: inhibitory control, cognitive flexibility, and working memory. Inhibitory control is the capacity to suppress goal-irrelevant stimuli and behaviors, encompassing both attentional inhibition and response inhibition (Tiego et al., 2018), including interference control (e.g., suppressing intrusive thoughts) and selective attention (Diamond, 2013). Working memory is characterised by the propensity to maintain and manipulate information (Baddeley, 1992). Cognitive flexibility refers to the capacity to adapt mental representations in response to changing environmental demands (Braem and Egner, 2018), and is closely related to task switching, defined as the shifting of attention between competing tasks or rules (Monsell, 2003). There is much evidence to suggest overlap among these components (Posner and Presti, 1987; Miyake et al., 2000; Mackie et al., 2013) organised as a set of mutually dependent functions entailing both unity (i.e., shared mechanisms supporting goal-directed action), and diversity (e.g., the distinct process that each function serves) with each function contributing a differentiated process within the broader executive system (Mackie et al., 2013; Miyake et al., 2000).

### 1.1 The effects of physical exercise on executive functions

In examining executive functions such as task switching and cognitive flexibility, researchers (Kamijo and Takeda, 2010; Davranche et al., 2015; McMorris, 2021) have reported differential effects of physical exercise on these processes. The influence of physical exercise has demonstrated significant effects on executive functions, with acute and chronic exercise yielding distinct effects [e.g., acute exercise improving task switching (Kamijo and Takeda, 2010), and chronic exercise improving long-term working memory in older adults (Zhidong et al., 2021; Yuan et al., 2023)]. While acute exercise refers to single bouts of physical activity (Basso and Suzuki, 2017), chronic exercise refers to a pattern of regular and repeated exercise over an extended period (e.g., weeks, months, years) (Chaire et al., 2020). Evidence suggests that both acute and chronic exercise elicits selective improvements in executive functions subserved by frontal-lobe-dependent processes (e.g., attention allocation, inhibitory control), consistent with the selective improvement hypothesis (Kramer and Colcombe, 2018; Smiley-Oyen et al., 2008; West, 2000; West, 1996) (e.g., greater benefits observed for task-switching or response inhibition compared to simple reaction time). Such benefits in executive functioning are particularly relevant in exercise contexts (Kramer and Colcombe, 2018), where these processes form a cognitive foundation for effortful control, supporting repeated goal-directed behaviour during physically demanding tasks (Audiffren and André, 2019).

### 1.2 Effortful control and dual-process theories

The effort hypothesis posits that the capacity to sustain health-related behaviours (e.g., regular participation in exercise), is contingent upon the engagement of executive functions. The aforementioned executive functions (e.g., working memory, inhibitory control, and cognitive flexibility) are foundational to effortful control, identified as crucial to maintaining adherence to physical exercise (Audiffren and André, 2019). Within this context, dual-process theories offer a complementary explanatory framework distinguishing between autonomous (Type 1) and controlled (Type 2) processing modes (Furley et al., 2015; Furley and Wood, 2016). These modes are differentiated by their reliance on attentional control and working memory resources (Furley and Wood, 2016), such that Type 2 processing reflects the deliberate, goal-directed operations underlying effortful control. Given that executive functions subserve effortful control and are activated during cognitively and physically demanding tasks, the repeated engagement of Type 2 processing in exercise contexts constitutes a mechanism through which these regulatory capacities may be strengthened – a premise consistent with the effort hypothesis (Audiffren and André, 2019).

### 1.3 The influence of music on executive functions and affective responses during exercise

Listening to music during physical exercise has been attributed to several mechanistic benefits related to executive functions (Terry et al., 2020). Such benefits include the redirection of attentional focus away from interoceptive cues, such as sensations of discomfort during physical exercise, resulting in reduced perceived exertion and improved allocation of cognitive resources toward goal-directed behaviour and attentional control under physical strain (Heath and Shukla, 2020; Hutchinson et al., 2018; Terry et al., 2020). This allocation of attention, whether internally directed (associative) or externally directed (dissociative), influences the extent to which cognitive resources are allocated toward executive functions in exercise contexts (Hutchinson et al., 2018; Terry et al., 2020). Rhythmic synchronisation has also been shown to improve temporal prediction and facilitate sustained attention during movement-based tasks (Karageorghis, 2016). The efficacy of these mechanisms appears contingent upon musical parameters, such as self-selected as well as tempo-appropriate music linked to reductions in cognitive load during physical exercise (Hutchinson et al., 2018; Hutchinson and Karageorghis, 2013; Karageorghis et al., 2021b). In parallel, music’s affective properties can be understood through the lens of the Circumplex Model of Affect (Ekkekakis and Petruzzello, 2000; Hardy and Rejeski, 1989), which frames core affect—defined as the fundamental neurophysiological state underlying emotional experiences—along two dimensions: affective valence (pleasant–unpleasant) and affective arousal (low–high energy). These dimensions are influenced by music as it is listened to during physical exercise. Specifically, moderate-to-fast-tempo (120–130 beats per minute, BPM), preference-based (e.g., self-selected) music may engender elevated affective valence and/or affective arousal, making it suitable for high-energy exercise (e.g., cycling at high intensities; see Karageorghis et al., 2021a, 2021b). Conversely, preference-based slower-tempo music (60–90 BPM) appears to reduce affective arousal while maintaining elevated affective valence, thereby eliciting recuperation during low-intensity activities (e.g., walking or light cycling as a form of post-exercise recovery; Terry et al., 2020). However, these effects appear to diminish at higher exercise intensities, where cognitive and physiological demands increase.

### 1.4 Moderating effects of exercise intensity on cognitive and affective responses

Low-to-moderate intensity physical activity (defined as 30–59% of VO₂ reserve, or activities such as brisk walking and light cycling, e.g., Garber et al., 2011) has been identified as a significant moderator of cognitive improvements, with activity within this range associated with improved performance on executive function measures (Chang et al., 2012; Davranche et al., 2015; Olson et al., 2016; Bergelt et al., 2024). In contrast, exercise performed at higher intensities (above ~70% of VO_2_ max) often yields more variable cognitive effects (Davranche et al., 2015), potentially due to individual differences in physiological and cognitive load (e.g., one’s tolerance for increased physical demands by regulating interoceptive processing during exercise). Similarly, there is a growing body of evidence showing the use of music at low-to-moderate exercise intensities significantly influencing executive function domains by minimizing interoceptive demands and elevating affective responses (Bigliassi et al., 2018, 2019; Hutchinson et al., 2018; Terry et al., 2020).

A salient explanation is Dual-Mode Theory (DMT) suggesting higher exercise intensities increase variability in cognitive and affective responses due to shifts between automatic (e.g., Type 1, immediate responses of pleasure or discomfort) and controlled processes (e.g., Type 2, deliberate behaviours, such as adhering to an exercise routine based on long-term goals) (Ekkekakis, 2003; Furley et al., 2015). DMT highlights the importance of the ventilatory threshold (VT) in determining affective and physiological responses. In the present review, exercise intensity is therefore treated as a key contextual moderator when interpreting music-exercise effects on executive functions and core affect, and we return to these mechanisms in the Discussion.

### 1.5 The present study

Given the described cognitive benefits of acute exercise and music, coupled with the likelihood of these being perceived as making the exercise experience more pleasant, there has been growing interest in the application of combined music and acute exercise protocols as a means to engage executive functioning (Ayaz et al., 2025; Satoh et al., 2014). Yet, to the authors’ knowledge, a review examining whether and how music and acute exercise may interact to influence executive functions and affective responses remains lacking. Considering how music and acute exercise interact in a systematic manner is enticing, as both exercise and music have been independently associated with cognitive and affective benefits [see, e.g., meta-analyses by Chang et al. (2012), Kramer and Colcombe (2018), and Terry et al. (2020)], thereby contributing to developing participant health and well-being interventions (Hars et al., 2014; Satoh et al., 2014). Accordingly, this review examines how music influences executive functions and affective responses during acute exercise by synthesizing findings from narrative syntheses and meta-analyses. Specifically, it is guided by two central research questions:

1. How has music listening been examined in relation to executive functions and affective responses during acute exercise?
2. Which executive function domains and affective outcomes have been targeted in the existing literature, and what patterns emerge regarding the direction and magnitude of music’s effects during exercise?

## 2 Materials and methods

In this systematic review, the primary comparison was between acute exercise with music listening compared to acute exercise without music listening in recreationally active adults aged 18 and over. This study was conducted and is reported in line with the Preferred Reporting Items for Systematic Reviews and Meta-Analyses (PRISMA) protocol (Page et al., 2021).

### 2.1 Eligibility criteria

We included studies that met the following criteria: The population comprised recreationally active individuals. Studies focusing on elite athletes, sedentary individuals, or those managing clinical conditions (e.g., psychiatric disorders) were excluded due to the distinct contextual and physiological characteristics of these groups (which may have limited exploring broader trends across the literature). This included males and females across different age ranges, with a focus on acute exercise. The intervention involved music listening during exercise, targeting at least one executive function of interest, such as attention allocation, inhibitory control, cognitive flexibility, working memory, or overall cognitive performance, and affective outcomes such as core affect. Executive function outcomes (e.g., inhibitory control, working memory) were specified as the primary outcomes of interest; affective responses were considered secondary and included only if reported in studies that also assessed executive function. The comparator condition was typically acute exercise alone, without music (with one exception; see Section 2.2). The outcomes assessed were music and acute exercise effects on executive functions and affective outcomes, specifically those listed in Table 1. The study design was limited to experimental designs (as these allow for controlled testing of the effects of music during exercise), including randomised controlled trials (RCTs) and crossover trials. Additionally, we included only articles published in English in peer-reviewed journals or as theses/dissertations, given the internal review processes associated with these formats.

**Table 1:** Operationalisations of outcomes of interest.

Outcome	Operational definition	Measurement tool(s)	Key references
Attention allocation	The process of directing focus towards internal sensations (association) and/or external stimuli (dissociation) during physical exercise.	Association-dissociation questionnaire; dual-task paradigms	Tammen (1996)
Inhibitory control	Suppressing impulsive actions (e.g., abrupt changes in intensity or technique) and resisting distractions from internal (e.g., negative thoughts) and external (e.g., environmental factors) sources. It supports deliberate decision-making and attention allocation during physical exercise	Stroop task, Go/No-Go Task	Diamond (2013) and Tiego et al. (2018)
Cognitive flexibility	Shifting attention between internal and external stimuli, modifying exercise behaviour based on feedback.	Cognitive flexibility inventory; task-switching paradigms	Diamond (2013) and Braem and Egner (2018)
Task switching	A component of cognitive flexibility involving attention allocation, to enable shifts between different cognitive and motor tasks. Efficiency and speed of task switching are influenced by task complexity, individual differences, and practice.	Task-switching paradigms; dual-task tests	Diamond (2013)
Working memory	Actively holds, manipulates, and processes information necessary for tasks like retaining instructions, monitoring feedback, and adapting to exercise behaviour.	N-back tasks; digit span tasks	Baddeley (1992) and Diamond (2013)
Overall cognitive performance	Broadly the ability to process and respond to information during exercise.	Mini-Mental State Examination (MMSE); cognitive load measures	Diamond (2013)
Core affect	Encompasses both emotions and moods, characterised by two dimensions: valence (pleasure-displeasure) and arousal (activation-sleepiness). Affect arises from physiological processes, cognitive appraisals, and situational influences and can range from basic reflexive responses to complex emotions.	Feeling Scale, Felt Arousal Scale Two-Dimensional Mood Scale	Hardy and Rejeski (1989)

### 2.2 Preregistration deviations

One exception was made during study selection: a study (see Chen et al., 2021) that did not include a true no-music control was retained, as a mismatched music condition (e.g., slower or faster tempo) was initially deemed a reasonable proxy. We acknowledge that such conditions may introduce distinct cognitive-affective effects rather than serving as a neutral baseline.

### 2.3 Information sources

We searched the following databases: (1) Web of Science, (2) SPORTDiscus, (3) MEDLINE, (4) Embase, (5) PubMed, (6) CINAHL Complete, (7) Cochrane Library, (8) Scopus databases, (9) PsycINFO, and (10) Google Scholar. The database search was supplemented by forward and backward snowball searches. The snowball search continued until no new sources could be identified. Specifically, the backward snowball search involved scanning the reference lists of all included articles for potential sources, while the forward snowball search identified additional studies by examining articles that cited the included studies. The initial inter-rater agreement for the identification of relevant sources was *k* = 0.88, indicating a strong level of agreement among the two individuals performing two independent snowball searches (AD, JV).

### 2.4 Search strategy

A literature search was performed using terminology related to executive functions being affected by music listening in physically active adults during exercise sessions. Specifically, the following search term was used: (TS = (“Executive functio*” OR “Inhibitory control” OR “Working memory” OR “Cognitive flexibility” OR “Task switching” OR “Attention” OR “Neurocognitive task*” OR “Goal-driven decision-making” OR “Dual-process theor*” OR “Autonomous processing” OR “Cognitive Control” OR “Controlled processing” OR “Ironic process* theory” OR “Affect”)) AND (TS = (“Music listening” OR “Music intervention*” OR “Music-based intervention” OR “Music and cognition”)) AND (TS = (“Physical exercise” OR “Sports performance” OR “Exercise-induced cognition” OR “Performance enhance*”)). The full search strategy can be found in the review registration document (PROSPERO CRD42023465958).

### 2.5 Selection process and data collection process

**Figure 1:**
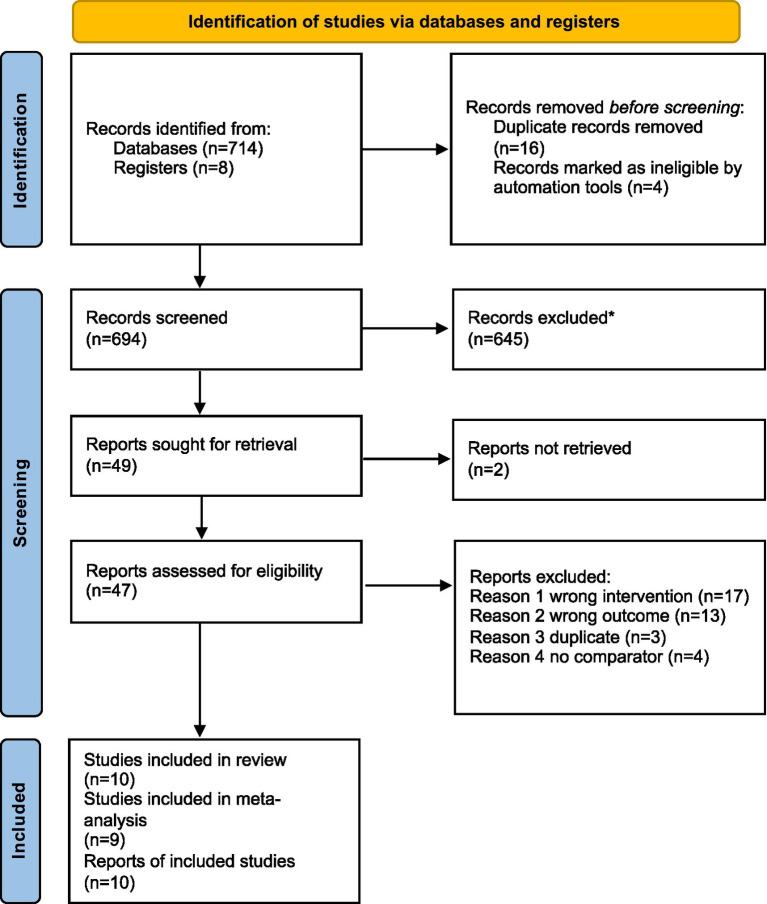
PRISMA information flow describing the screening process. *All records excluded by ASReview (van de Schoot et al., 2021).

The citations of all retrieved articles were imported into Zotero and all duplicates were manually removed. Study title and abstract were then screened by three authors (AD, JV, JSB) using ASREVIEW (van de Schoot et al., 2021). If the article could not be excluded on the basis of the title or abstract, the retrieved full-text articles were then assessed for inclusion by two authors independently (AD, JV). At each stage, disagreements were resolved through discussion with a third author (JSB), who acted as a referee when consensus could not be reached between the initial screeners (Figure 1).

### 2.6 Data extraction

The studies’ information was extracted to a spreadsheet. This included study characteristics, such as the targeted executive functions, the study design, cognitive process measurements, the music protocol, and study outcomes. Where available, quantitative data suitable for meta-analysis (e.g., mean values, standard deviations, or effect sizes for executive function outcomes) were also extracted. In instances where necessary data for meta-analysis were not reported, corresponding study authors were contacted to obtain the missing data for analysis.

### 2.7 Study risk of Bias assessment

**Figure 2:**
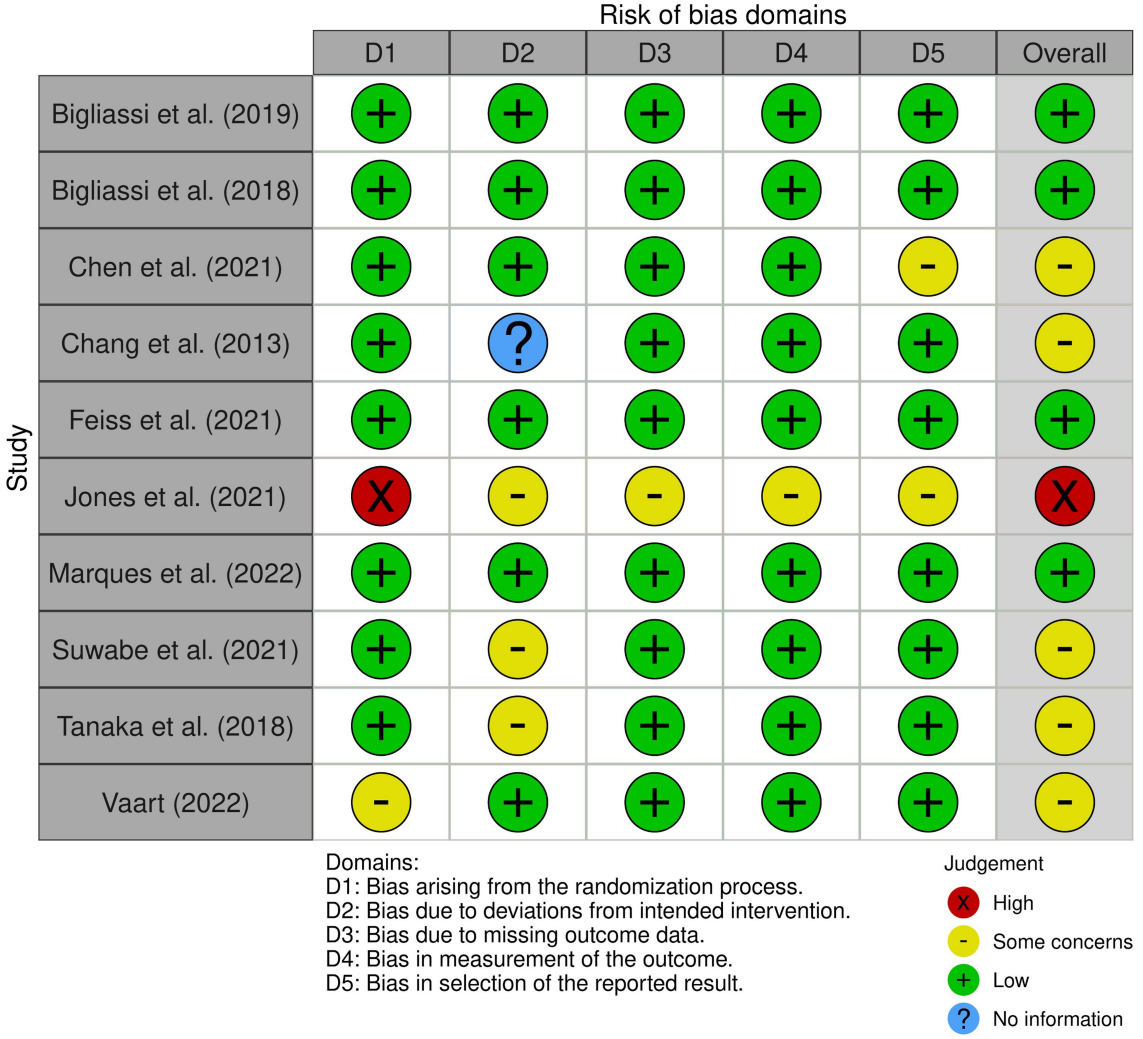
Evaluation of risk of bias in the studies included in the review, categorised across five domains from D1 to D5. An overall bias risk assessment for each study is also provided, summarising the findings across all domains.

The quality of the studies were assessed by two authors (AD, JV) using the Joanna Briggs Institute critical appraisal checklist (JBI, Grammatopoulos et al., 2023), including tools for Quasi-Experimental Appraisal, and the Revised Checklist for RCTs (Figure 2). The JBI critical appraisal tools were chosen for their adaptability to diverse study designs, providing a structured and standardised approach to assessing risk of bias. While alternative tools such as the Cochrane framework are commonly used in clinical reviews, the JBI tools were more suitable for the interdisciplinary nature of this review, encompassing experimental studies in sports and cognitive psychology.

### 2.8 Operationalisation of outcomes of interest

The main outcomes of interest in the review are operationalised in the following table (Table 1). It contains the cognitive process as the outcome of interest (see “outcome”), an “operational definition” of it, the “measurement tool(s)” and “key references” associated with the operationalisation (see, e.g., Appendix A for a full description of the operationalisation of outcomes of interest in this review).

### 2.9 Data synthesis and analysis methods

We conducted a narrative synthesis to qualitatively analyse findings across four identified executive functions: attention allocation, inhibitory control, cognitive flexibility and working memory. All affective outcomes were analysed under core affect. Studies were categorised by their primary outcomes, and trends were identified by examining the direction and magnitude of effects, alongside variations in study characteristics, such as participant demographics, exercise intensity, music conditions (including both synchronous and asynchronous conditions), and methodological designs. Heterogeneity was addressed by highlighting contextual factors, including differences in exercise protocols (e.g., exercise intensity, duration, modality, task type) and music conditions (e.g., tempo-matched, self-selected music).

To examine how music listening may exert an effect on executive function outcomes, we performed a meta-analysis of the outcomes of interest in eligible studies. Eligible studies included those that compared music listening with acute exercise, to acute exercise without music listening, with both a control group and an intervention arm targeting the outcomes of interest. Additionally, a minimum of three studies was required to provide suitable, quantifiable data for inclusion in any single meta-analytic cluster [in line with recommendations to ensure stability of pooled estimates and enable basic heterogeneity assessment (Higgins and Green, 2008; The Campbell Collaboration, 2014)]. Exploratory subgroup analyses (e.g., affective valence vs. arousal) were conducted when at least three studies contributed relevant outcomes.

For quantitative synthesis, we calculated Hedges’ *g* effect sizes and standard errors using the tool developed by Wilson (2023). Where multiple values were extracted for the same outcome, weighted averages were calculated to standardise effect sizes. These standardised values were analysed using random-effects models estimated with REML. Heterogeneity was assessed using the *I*^2^ statistic, *τ*^2^ statistic, and *Cochran’s Q statistic*. By reporting *I*^2^, *τ*^2^, and *Cochran’s Q statistic* we provide a comprehensive assessment of heterogeneity, capturing absolute variance (*τ*^2^), relative proportion of variability attributable to heterogeneity (*I*^2^), and a formal test of the homogeneity assumption (*Q*). To account for the small number of studies in several comparisons (low k), we applied the Hartung–Knapp–Sidik–Jonkman (HKSJ) adjustment to provide more accurate confidence intervals and control Type I error rates, as recommended for meta-analyses with small sample sizes (see, e.g., Higgins et al., 2019).

We then conducted a meta-regression analysis to examine whether study-level variables – such as sample size, mean participant age, exercise intensity, music tempo and the use of self-selected or researcher-selected music – moderated the effect sizes of executive function outcomes when combining music with acute exercise, compared to acute exercise alone. A random-effects meta-regression model (REML with HKSJ adjustment) was used to assess the independent contribution of each moderator while accounting for between-study heterogeneity. Finally, a descriptive subgroup analysis based on exercise intensity (low, moderate, high) was conducted using one effect size per study to explore potential patterns. This analysis was intended to characterise possible directional differences. Data and syntax files for these analyses are available as Supplementary Files (e.g., OSF, doi: 10.17605/OSF.IO/XFN3M).

## 3 Results

### 3.1 Study selection

The initial search identified 714 articles. After removing 16 duplicates and four ineligible articles, 645 were excluded based on title and abstract for not meeting inclusion criteria. Forty-nine full-text articles were sought for retrieval; two could not be accessed (no response from authors), leaving 47 articles to be assessed for eligibility. Thirty-seven articles were excluded for not using the desired intervention, lacking relevant outcomes, turning out to be secondary reports of other identified studies, and reporting no comparator. In total, 10 articles were deemed eligible for inclusion in the review (Figure 1). As the meta-analytic approach required at least three studies per outcome cluster, and there was insufficient data for certain identified outcomes, such as working memory (e.g., only two studies reported data for this outcome), nine articles were included in the meta-analysis.

### 3.2 Study characteristics

The study characteristics (Table 2) include a diverse set of studies conducted in countries such as the United Kingdom (UK) (*n* = 2), Brazil (*n* = 1), Korea (*n* = 1), China (*n* = 1), the United States of America (USA) (*n* = 2), Japan (*n* = 2), and Canada (*n* = 1). These studies target a wide range of populations, including healthy young adults, and university students. A variety of study designs are used, such as RCTs, crossover designs, and repeated measures designs. Similarly, the included studies varied in music and exercise protocols, encompassing asynchronous and synchronous music, and low-, moderate-, and high-intensity exercise.

**Table 2:** Characteristics of reviewed studies.

Reference	Country	Age y	Sample size N	Population	Study design	Music protocol	Exercise protocol and intensity	Executive function measurement	Outcome measurement timepoint	Target executive function	*g* [95% CI] of executive function and/or affective response
Bigliassi et al. (2019)	UK	24.20 ± 4.9	19	Healthy Adults	Block-design	Music listening (headphones) Researcher-selected (119 BPM) Asynchronous music	Handgrip task (interval) Moderate exercise intensity	Attention scale FAS^a^	Pre, post	Attention allocation Core affect	*g* = 0.86 (95% CI: [−0.10, 1.62]) for Attention allocation *g* = 0.75 (95% CI: [0.10, 1.39]) for Core affect
Bigliassi et al. (2018)	UK	23.5 ± 4.3	24 (11 women, 13 men)	Healthy adults	Within-subjects repeated measures	Music listening (earphones) Researcher-selected (160 BPM) Asynchronous music	Self-paced light-intensity outdoor walking (continuous) Low exercise intensity	Attention scale FAS	Pre, post	Attention allocation Core affect	*g* = 3.05 (95% CI [2.22, 3.87]) for attention allocation *g* = 3.75 (95% CI: [2.81, 4.68]) for core affect
Chang et al. (2013)	Korea	21.1 ± 1.5	28 females	Healthy university students	Four-condition crossover (rest, high-decibel music only, exercise only, exercise and high-decibel music)	High-decibel music (≥100 dB; laboratory speakers) N/A	Cycling at 70–75% VO₂ max (continuous) High exercise intensity	Stroop task	Pre, post	Inhibitory control	Outcomes N/A
Chen et al. (2021)	China	21.54 ± 2.26	90 (45 females, 45 males)	Young adults	RCT	Music listening (earphones) Researcher-selected (SM^b^ 60–65 BPM; M 120–140 BPM; FM 155–165 BPM) Synchronous and Asynchronous Music	30-min moderate-intensity cycling with music (continuous) Moderate Exercise Intensity	Stroop Task n-back Test n-back test, M-OSTi^c^ CMAC^d^	Pre, post	Inhibitory control Cognitive flexibility Working memory Core affect	*g* = 0.97 (95% CI: [0.50, 1.44]) for inhibitory control Outcomes N/A Outcomes N/A *g* = 0.26 (95% CI: [0.05, 0.46]) for core affect
Feiss et al. (2021)	USA	25.0 ± 4.0	63	Young adults	Between-subjects, randomised controlled design	Music listening (headphones) Researcher-selected (fast tempo 120 BPM; slow tempo 90 BPM; billboard hot 100 songs, past 6 months, multi-genre) asynchronous music	Isometric holds to exhaustion (continuous) Moderate exercise intensity	Attention Scale Affect grid	Pre, post	Attention allocation Core affect	*g* = −0.07 (95% CI [−0.27, 0.13]) for attention allocation *g* = 0.00 (95% CI [−0.20, 0.20]) for Core Affect
Jones et al. (2021)	USA	20.3 ± 1.7	12 (7 males, 5 females)	Undergraduate students	Randomised controlled pilot study	Music listening (laboratory speakers) Researcher-selected (sedative *M* = 69.85 BPM; Stimulative *M* = 148BPM) Asynchronous music	12-min self-paced HIIT workout (interval) High exercise intensity	Stroop Test	Pre, post	Attention allocation	*g* = 0.25 (95% CI: [−0.30, 0.80]) for attention allocation
Marques et al. (2022)	Brazil	27.0 ± 3.9	16	Adults	Randomised counterbalanced crossover design	Music listening (headphones) Researcher and self-selected music^e^ (140–160 BPM) Asynchronous music	Sprint intervals: 8 × 15 s all-out efforts (interval) High exercise intensity	Attention scale Feeling scale	Pre, post	Attention allocation Core affect	*g* = 0.62 (95% CI [0.22, 1.03]) for attention allocation *g* = −0.04 (95% CI [−0.43, 0.35]) for core affect
Suwabe et al. (2021)	Japan	20.9 ± 2.4	33 (21 males, 12 females)	Healthy young adults	Within-subject crossover design	Music listening (earphones) Self-selected (pre-approved researcher-provided playlists; 120 BPM) Synchronous music	10-min moderate intensity cycling bouts (continuous) Moderate exercise intensity	CWST^f^ Two-dimensional mood scale	Pre, post	Inhibitory control Core affect	*g* = 0.99 (95% CI: [0.48, 1.49]) for inhibitory control *g* = 2.28 (95% CI: [1.67, 2.90]) for core affect
Tanaka et al. (2018)	Japan	22.9 ± 0.5	15	Healthy young men	Randomised controlled crossover study	Music listening (headphones) Researcher-selected (~160 BPM) Asynchronous music	20-min moderate-intensity cycling (60% VO₂ peak) (continuous) Moderate exercise intensity	Stroop task FAS	Pre, post, +10/20/30 min	Inhibitory control Core affect	*g* = 2.68 (95% CI: [1.70, 3.65]) for inhibitory control *g* = −0.32 (95% CI: [−1.03, 0.38]) for core affect
Vaart (2022)	Canada	23.4 ± 2.5	24 (14 females, 10 males)	Healthy young adults	Repeated measures design	Music listening (headphones) Researcher-selected (Classical music playlist, 120–140BPM) Synchronous and asynchronous music	30-min moderate-intensity recumbent cycling (continuous) Moderate exercise intensity	Stroop task Reverse Corsi Block Task	Pre, post	Inhibitory Control Working Memory	*g* = −0.05 (95% CI: [−0.63, 0.52]) for inhibitory control Outcomes N/A

a Felt Arousal Scale (FAS).

b Slower mismatched music 60–65 BPM; matched music 120–140 BPM; faster mismatched 155–165 BPM.

c More-odd shifting task (M-OST).

d Chinese mood adjective checklist (CMAC).

e Participants created playlists with preferred music, but a pre-curated Spotify playlist (“power workout”) was randomly selected by researchers for the exercise session.

f Color-Word Stroop Task (CWST).

### 3.3 Identification of outcomes and reported measurements of outcomes across studies

The identified outcomes of interest across the studies are: core affect (*n* = 7), inhibitory control (*n* = 6; five of which contributed data to meta-analysis), attention allocation (*n* = 4), working memory (*n* = 2), and cognitive flexibility (*n* = 1) (see Table 3). No outcomes were reported for task switching or overall cognitive performance. Interrater reliability was assessed for the identification of executive function outcomes across the studies, and was found to be acceptable, *k* = 0.667, indicating a moderate to substantial level of agreement among the two raters. Even though initial disagreements were resolved, the moderate reliability demands caution when interpreting the analysis of the identified outcomes of interest.

**Table 3:** Outcomes of interest identified across the included studies.

Reference	Attention allocation	Inhibitory control	Cognitive flexibility	Working memory	Core affect
Bigliassi et al. (2019)	•				•
Bigliassi et al. (2018)	•				•
Chang et al. (2013)		•			
Chen et al. (2021)		•	•	•	•
Feiss et al. (2021)	•				•
Jones et al. (2021)		•			
Marques et al. (2022)	•				•
Suwabe et al. (2021)		•			•
Tanaka et al. (2018)		•			•
Vaart (2022)		•		•	

We categorised the studies based on specific executive functions and measurement tools. Studies under attention allocation (Bigliassi et al., 2018, 2019; Chen et al., 2021; Marques et al., 2022), used Tammen’s single-item attention scale (Tammen, 1996) to measure associative and dissociative attention. Inhibitory control was assessed using variations of the Stroop task to measure reaction times and error rates in studies by Chang et al. (2013), Tanaka et al. (2018), Chen et al. (2021), Jones et al. (2021), Suwabe et al. (2021), and Vaart (2022). Cognitive flexibility was examined using the n-back task by Chen et al. (2021). Similarly, working memory was assessed by Chen et al. (2021) and Vaart (2022), with Chen et al. (2021) using the n-back (1-back and 2-back) and More-odd Shifting Task (M-OST) task, and Vaart (2022) employing the Reverse Corsi Block Task to examine visuospatial working memory. Core affect was assessed by Tanaka et al. (2018), Bigliassi et al. (2018, 2019), Chen et al. (2021), Feiss et al. (2021), Suwabe et al. (2021), and Marques et al. (2022), who used the Feeling Scale (FS, Hardy and Rejeski, 1989), Felt Arousal Scale (FAS, Svebak and Murgatroyd, 1985), Affect Grid (AG, Russell et al., 1989), Chinese Adjective Mood Checklist (CMAC, Chen et al., 2021) and Two-Dimensional Mood Scale (TDMS, Suwabe et al., 2021) to measure affective outcomes. These measures, with the exception of CMAC, have been commonly used in sports and exercise research domains, with high reliability and validity among them (Terry et al., 2020). All studies, with the exception of Tanaka et al. (2018), recorded outcomes from their measurements immediately before and after exercise. Tanaka et al. (2018) measured inhibitory control outcomes before exercise, immediately after exercise, and at three intervals (10, 20, and 30 min) during the post-exercise recovery period. Table 4 summarises the measurement tools used across the included studies to assess executive function and affective outcomes. Each tool is linked to specific constructs with key references.

**Table 4:** Summary of measurement outcomes for executive function and affective outcomes.

Outcome	Measurement tool	Description	Key references
Attention allocation	Tammen’s attention scale	Measures associative (internal focus on sensations, e.g., breathing) and dissociative (external focus, e.g., music) attentional strategies.	Tammen (1996)
	Stroop task	Assesses selective attention by requiring attention on ink color while suppressing the automatic response of reading the word.	Zysset et al. (2001)
	N-back test	Evaluates temporal sequence monitoring and response inhibition, incorporating lure trials to measure attention and working memory.	Kane et al. (2007)
Inhibitory control	Stroop task	Evaluates ability to suppress automatic responses and regulate behaviour.	Zysset et al. (2001)
	CWST (Color Word Stroop Test)	Assesses inhibitory control through suppression of irrelevant information while responding to task demands.	Zysset et al. (2001)
Cognitive flexibility	More-odd Shifting Task (M-OST)	Requires task-switching and adaptability to changing demands, including memory updating and inhibition of irrelevant information.	Chen et al. (2014)
Task switching	Not identified	Not identified	N/A
Working memory	N-back Test	A continuous-recognition task requiring maintenance and updating of dynamic rehearsal sets.	Kane et al. (2007)
	Reverse Corsi Block Task	Measures visuospatial memory by recalling spatial sequences in reverse order.	Vandierendonck et al. (2004)
Overall cognitive performance	Not identified.	Not identified.	N/A
Core affect	Feeling Scale (FS) & Felt Arousal Scale (FAS)	Assesses affective valence (FS) and arousal (FAS) during physical exercise.	Hardy and Rejeski (1989)
	Two-Dimensional Mood Scale (TDMS)	Measures mood along pleasure-displeasure and arousal dimensions.	Schubert (1999)
	Chinese Mood Adjective Checklist (CMAC)	Captures diverse emotional responses using descriptive adjectives.	Chen et al. (2021)

Several tasks (e.g., Stroop, n-back) load on multiple executive components; for meta-analytic clustering, studies were assigned based on the primary construct and dependent variable emphasised (see Section 3.4).

### 3.4 Study categorisation into meta-analysis clusters

As the Stroop task is not a pure measure of either inhibitory control or attention allocation (Hagyard et al., 2021), we differentiated between studies focusing on inhibitory control and those examining attention allocation, specifically organizing them into two distinct meta-analysis clusters. For attention allocation, studies were required to use a variation of the Stroop task as a measure of selective attention, emphasizing reaction times, particularly the Stroop effect (i.e., the difference between incongruent and congruent trial times), as the primary dependent variable. These studies needed to explicitly state their aim of investigating attention allocation or selective attention in the Stroop context. For inhibitory control, studies also had to employ the Stroop task but emphasise Stroop interference scores (which quantify the suppression of participants’ automatic responses, Hagyard et al., 2021). These studies had to clearly indicate a focus on inhibitory control or prepotent response suppression within the Stroop paradigm. Inclusion to this cluster was extended to studies incorporating other inhibitory control tasks, such as the Flanker or Go/No-Go tasks. Core Affect was also included as a separate cluster. Following the Circumplex Model of Affect (Hardy and Rejeski, 1989), we categorised affective responses engendered by music along two dimensions: valence, which represents the spectrum of pleasure to displeasure, and arousal, which entails the level of activation or energy.

### 3.5 Risk of bias in studies

Following the assessment of the study quality using the JBI critical appraisal checklist tools (Grammatopoulos et al., 2023), the nine criteria were adapted to the five risk of bias domains found in the McGuinness and Higgins (2021) *R* package for risk-of-bias assessments (robvis). This assessment tool evaluates the risk of bias resulting from the randomisation process (D1), deviations from intended intervention (D2), missing outcome data (D3), measurement of the outcome (D4), and selection of the reported result (D5). Each domain is assessed using a judgement scale indicating high risk of bias (red cross), some concerns (yellow circle), low risk of bias (green plus), and No Information (blue question mark), with a summary of the overall risk of bias for each study presented in Figure 2. The overall risk of bias rating was determined by assigning the lowest rating observed across any of the five domains (i.e., if a high risk was present in any domain, the overall rating was also high).

**Figure 3:**
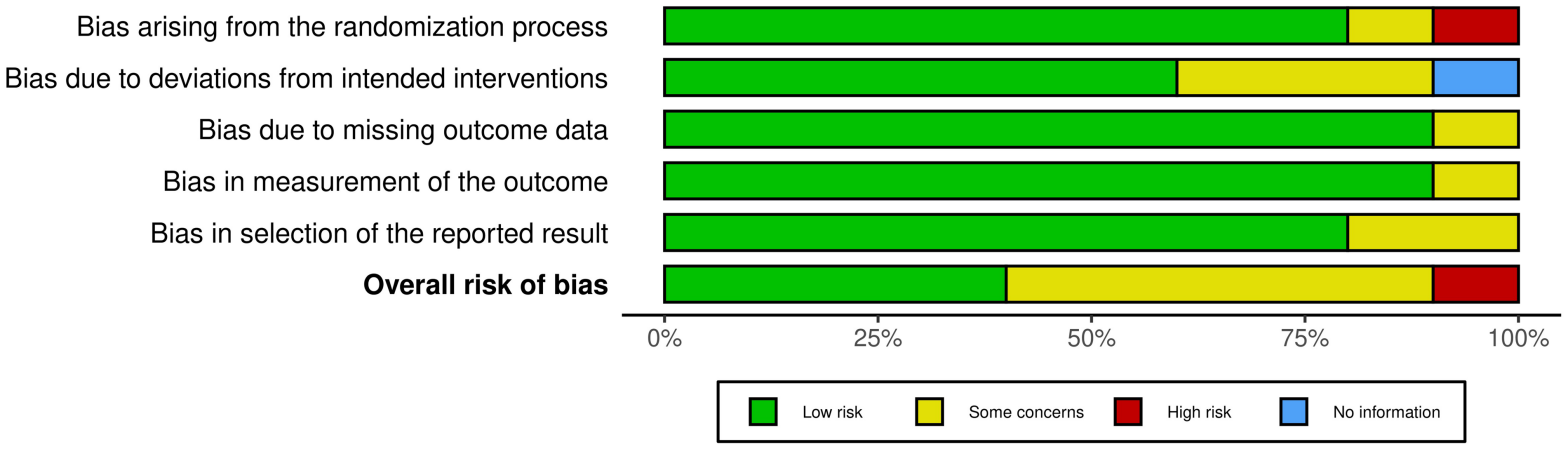
The overall risk of bias for each study is also summarised, with green representing low risk, yellow indicating some concerns and red representing high risk. The majority of studies fall within low risk for most domains, though some domains exhibit higher proportions of “some concerns” for bias.

Of the 10 studies, four studies were rated for a low risk of bias. Five studies received a moderate (some concerns) rating, and one study received a high rating in risk of bias. We included all studies in the review regardless of their quality rating (Figures 2, 3).

### 3.6 Executive function and core affect outcomes

Individual study findings were analysed qualitatively across the following outcomes of interest: attention allocation, inhibitory control, cognitive flexibility, working memory, and core affect. A single overall meta-analysis was not conducted due to substantial heterogeneity across datasets and outcomes. Instead, outcomes were categorised into distinct domains for separate analyses. For attention allocation (*n* = 4), inhibitory control (*n* = 5), and core affect (*n* = 7), both narrative and meta-analytic findings are reported within each subsection. Meta-analyses could not be conducted for cognitive flexibility (*n* = 1) and working memory (*n* = 2) due to the limited number of studies and available data for these executive functions.

#### 3.6.1 Attention allocation

##### 3.6.1.1 Narrative findings

Music was associated with shifts toward dissociative attention immediately post-exercise, particularly following low-to-moderate intensity bouts. However, the effectiveness of music to influence attention varied depending on study protocols and exercise modalities. Bigliassi et al. (2019) studied a sample of recreationally active adults performing isometric handgrip tasks (interval) at moderate intensities, and found that music assisted with subjective dissociative attention following exercise. Notably, Feiss et al. (2021) found that both fast- and slow-tempo music supported dissociative attention following a wall-sit exercise but not after a plank hold, indicating that the influence of music varies across isometric tasks. During sprint interval training (SIT), Marques et al. (2022) reported that music improved dissociative attention after recovery periods but had no significant effect following bouts of high intensity exercise, indicating music’s conditional utility (e.g., music may aid recuperation and recovery after low-intensity exercise phases, but is less effective in diverting attention allocation post high exercise intensities). Collectively, the evidence suggests that music might facilitate dissociative attention following moderate-intensity dynamic tasks (e.g., cycling) or recovery phases (low intensity exercise), but its effects may diminish after static (e.g., plank hold exercise) or high-intensity conditions, where associative attention may dominate.

##### 3.6.1.2 Meta-analytic findings

**Figure 4:**
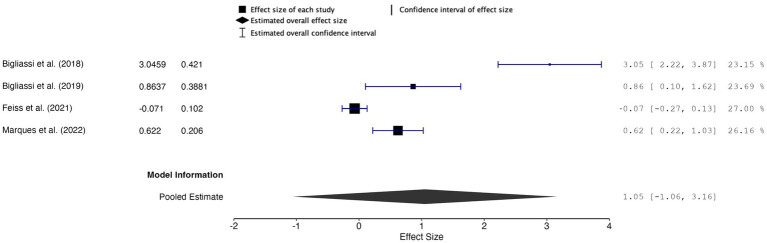
Forest plot of effect sizes for attention allocation outcomes of music during acute exercise compared to no music during acute exercise.

The overall effect size was 1.05, with a 95% confidence interval (CI) of −1.06 to 3.16 and a *p*-value of 0.210 (*k* = 4, *n* = 122) (Figure 4). These results were not statistically significant, indicating insufficient evidence to support a significant effect of music used during acute exercise on attention allocation. The random-effects model revealed high heterogeneity between studies, indicating substantial variability across the included studies (*Q* = 59.732, *p* < 0.001, *I^2^* = 94.98%, *τ*^2^ = 0.992). Furthermore, the 95% prediction interval ranged from −2.75 to 4.86, indicating the wide variability and uncertainty in the true effects that could be observed in future studies.

#### 3.6.2 Inhibitory control

##### 3.6.2.1 Narrative findings

Mixed results on inhibitory control were found as music was combined with acute exercise, with outcomes influenced by exercise intensity, protocol design, and the presence of auditory-motor coupling. Chen et al. (2021) found that tempo-matched music synchronised to participants’ heart rates during moderate-intensity aerobic exercise, significantly influenced post exercise inhibitory control, potentially due to auditory-motor coupling (e.g., participants exhibited improved Stroop task performance when cycling at a tempo aligned with music at 120–140 BPM, facilitating synchronisation between their heart rate and the auditory rhythm). Chang et al. (2013) examined high-intensity cycling (at 70–75% VO₂max) in healthy female university students under four conditions (rest, high-decibel music alone, exercise alone, and exercise with high-decibel music). High-intensity exercise improved Stroop performance relative to rest and music-alone conditions, but adding high-decibel music (~100 dB) did not confer additional behavioural benefits beyond exercise alone and was associated with reduced prefrontal oxygenation compared with exercise-only. This pattern suggests that exercise itself improved inhibitory control, whereas concurrently presenting high-decibel music did not provide incremental cognitive benefits and may even have introduced adverse physiological effects. Similarly, null results of music-exercise modalities were reported in Jones et al. (2021) and Suwabe et al. (2021). Specifically, Suwabe et al. (2021) report no significant differences between music and metronome conditions on inhibitory control following moderate-intensity cycling, while Jones et al. (2021) observed no such effects of music or high-intensity interval training on inhibitory control, suggesting that task sensitivity and protocol alignment (e.g., matching music tempo to exercise intensity) may be more salient to influence this outcome. Variability in participant characteristics further contributed to inconsistencies, with Chen et al. (2021) recruiting a larger, more diverse sample compared to smaller, demographically specific cohorts in Chang et al. (2013) and Jones et al. (2021). The mixed evidence indicates that music’s effects on inhibitory control might vary due to contextual factors such as task design and exercise intensity.

##### 3.6.2.2 Meta-analytic findings

**Figure 5:**
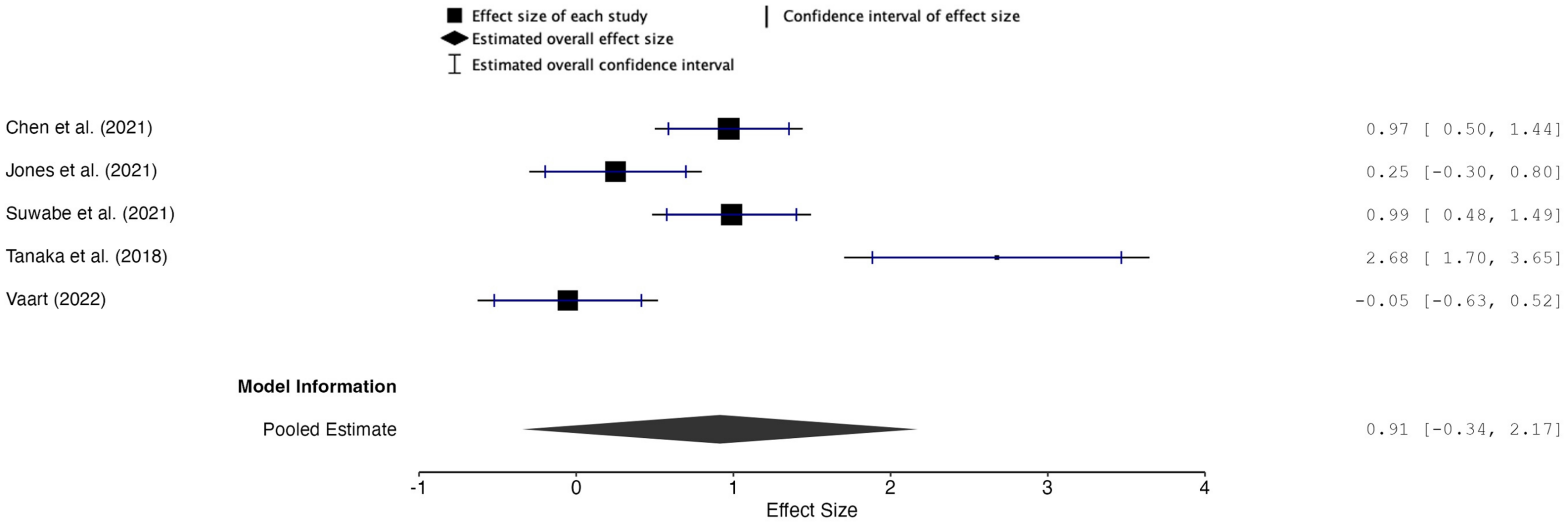
Forest plot of effect sizes for inhibitory control outcomes of music during acute exercise compared to no music during acute exercise.

The overall effect size for inhibitory control was *g* = 0.91, with a 95% confidence interval of −0.34 to 2.17 and a *p*-value of 0.114 (*k* = 5, *n* = 174), indicating insufficient evidence to support a reliable effect of music used during acute exercise on inhibitory control (Figure 5). The random-effects model revealed substantial between-study heterogeneity (*Q* = 27.51, df = 4, *p* < 0.001; *I*^2^ ≈ 85.5%, *τ*^2^ = 0.85), and the 95% prediction interval ranged from −1.93 to 3.76, indicating that true effects in future studies could plausibly range from small negative to large positive values.

#### 3.6.3 Cognitive flexibility

##### 3.6.3.1 Narrative findings

One RCT by Chen et al. (2021) examined cognitive flexibility using a combined music (i.e., pop music without lyrics, with three different tempi, 60–65 BPM, 120–140 BPM and 155–165 BPM) and exercise protocol (i.e., a 20-min bout of moderate-intensity aerobic exercise on a bicycle ergometer at 50–60 revolutions per minute, RPM) with 90 young adults. Results indicated no significant differences in cognitive flexibility outcomes across groups, suggesting that tempo variations did not critically influence this outcome.

#### 3.6.4 Working memory

##### 3.6.4.1 Narrative findings

Chen et al. (2021) found that music matched in tempo (120–140 BPM) with the exercise routine significantly improved working memory outcomes in 30 young adults, compared to slower and faster music tempi. In contrast, Vaart (2022) found no significant improvements in working memory between exercise (a 30-min session on a recumbent cycle ergometer at a moderate intensity, 55% of the participant’s heart rate reserve) alone and exercise with music (a musical playlist consisting of classical songs with a tempo ranging from 120–140 BPM) in 24 young adults.

#### 3.6.5 Core affect

##### 3.6.5.1 Narrative findings

Bigliassi et al. (2018) demonstrated that music significantly elevated affective arousal and produced more pleasurable participant experiences after light-to-moderate intensity exercise. Similarly, Chen et al. (2021) report that tempo-matched music – a form of sensorimotor synchronisation (e.g., Van Dyck et al., 2015) elevated affective valence and emotional states post moderate-intensity aerobic exercise, where synchronised music tempo and participant physiological rhythms (e.g., heart rate) improved the effects of music on core affect outcomes, compared to slower or faster-matched music.

Exercise intensity emerged as a potential variable moderating these outcomes: while tempo-matched music was particularly effective following sustained moderate-intensity efforts (Bigliassi et al., 2018; Chen et al., 2021), its effects were less pronounced after short, high-intensity sprints or isometric tasks (Feiss et al., 2021; Marques et al., 2022), where those physical demands may have outweighed music’s influence on cognitive and affective outcomes. Notably, Feiss et al. (2021) and Marques et al. (2022) reported no elevation in affective valence or arousal post high-intensity or isometric exercises, likely due to the dominance of physiological stressors (e.g., physical exertion), which potentially diminished music’s effectiveness under high physical demand. Overall, the affective responses (e.g., core affect) to music and acute exercise evident in the narrative synthesis indicate a context-dependent influence on core affect outcomes, elevating core affect at lower and moderate exercise intensities (e.g., tempo-matched music during steady-state aerobic exercise) but yielding inconsistent effects following high-intensity bouts or physiologically demanding exercise protocols (e.g., sprint interval training or isometric tasks).

##### 3.6.5.2 Meta-analytic findings

**Figure 6:**
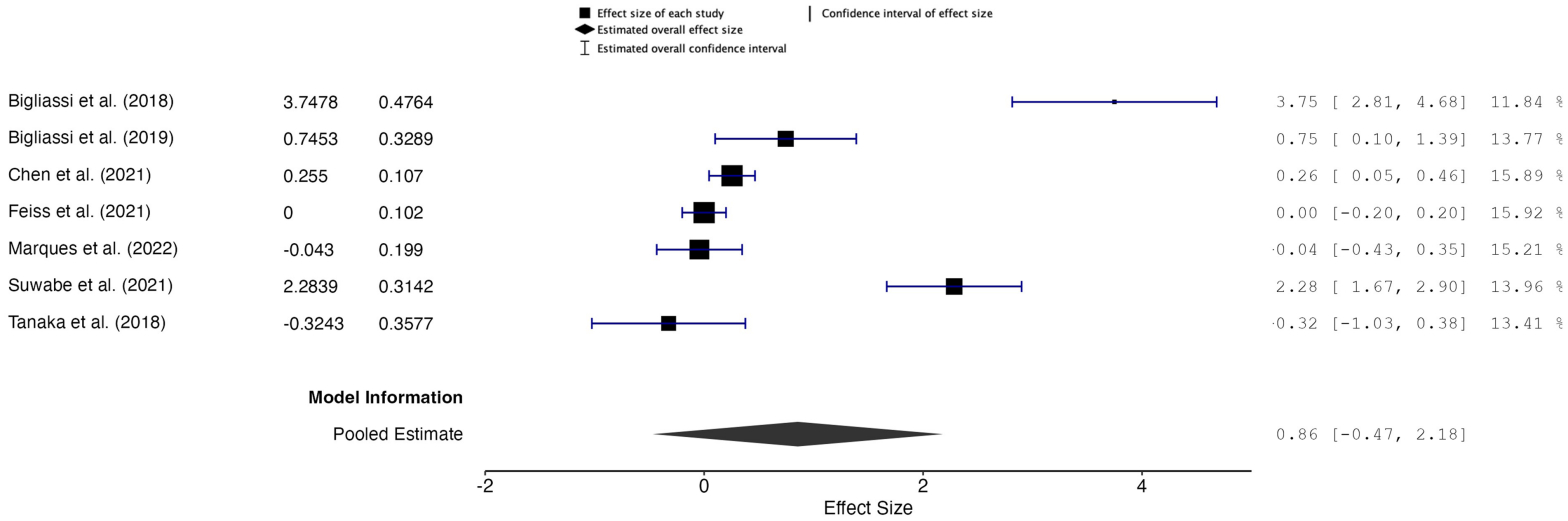
Forest plot of effect sizes for core affect outcomes of music during acute exercise compared to no music during acute exercise.

The overall effect size was 0.86, with a CI of −0.47 to 2.18 and a *p*-value of 0.17 (*k* = 7, *n* = 200), indicating that the results were not statistically significant, suggesting insufficient evidence to support a significant effect of music used during acute exercise on core affect outcomes (Figure 6). The random-effects model indicated high heterogeneity across studies (*Q* = 108.724, *p* < 0.001, *I^2^* = 94.48%, *τ*^2^ = 0.618), suggesting considerable variability between the studies. Additionally, the 95% prediction interval ranged from −1.48 to 3.19, highlighting that future studies may observe a wide range of effects.

A sensitivity analysis, which excluded one study by Bigliassi et al. (2018) due to its large effect size (*g* = 3.75), produced an overall effect size of 0.45, with a CI of −0.04 to 0.94 and a *p*-value of 0.228 (*k* = 6, *n* = 176). This result was also not statistically significant. The random-effects model indicated high residual heterogeneity (𝑄 = 54.476, *p* < 0.001, *I^2^* = 90.82%, *τ*^2^ = 0.308), suggesting considerable variability between the studies even after removing the outlier. The 95% prediction interval ranged from −0.093 to 0.993, indicating that the true effect sizes in future studies may vary widely.

##### 3.6.5.3 Subgroup analysis results: affective arousal

Given that core affect comprises valence and arousal dimensions, we further examined affective arousal and affective valence in subgroup analyses. The overall effect size was 1.37, with a CI of −3.85 to 6.58 and a *p*-value of 0.377 (*k* = 3, *n* = 58). This result was not statistically significant, suggesting insufficient evidence to support a significant effect of music used during acute exercise on affective arousal outcomes. The random-effects model indicated substantial heterogeneity (𝑄 = 47.558, *p* < 0.001, *I^2^* = 95.80%, *τ*^2^ = 3.317), highlighting significant variability across studies. The 95% prediction interval ranged from −8.048 to 10.781, indicating a wide range of potential true effect sizes in future studies.

##### 3.6.5.4 Subgroup analysis results: affective valence

The overall effect size was 0.79, with a CI of −2.30 to 3.88 and a *p*-value of 0.386 (*k* = 3, *n* = 79). This result was not statistically significant, suggesting insufficient evidence to support a significant effect of music used during acute exercise on affective valence outcomes. The random-effects model indicated substantial heterogeneity (Q = 42.557, *p* < 0.001, *I^2^* = 95.30%, *τ*^2^ = 0.744), highlighting significant variability across studies. Furthermore, the 95% prediction interval ranged from −4.040 to 5.621, indicating a wide range of potential true effect sizes in future studies.

### 3.7 Moderators of effects

Heterogeneity was identified in all meta-analyses. Therefore, a meta-regression analysis was performed to explore mean participant age, exercise intensity, and music tempo and the use of self-selected or researcher-selected music (music selection) as potential moderators of effect sizes, comparing combined music and exercise protocols with exercise alone. In our meta-regression analysis, exercise intensity, music tempo and the use of self-selected music or researcher-selected music (music selection) were coded as ordinal variables (1–3 for intensity and tempo; 1–2 for music selection). In the primary multivariable model, these were entered as numeric predictors (testing linear trends). In an exploratory follow-up, exercise intensity was also examined as a three-level categorical moderator. The analysis encompassed all outcomes of interest due to the small number of studies available for meta-analyses.

#### 3.7.1 Meta-regression analysis

The multivariable meta-regression pooled 18 effect sizes from nine studies and examined all moderators simultaneously (Table 5). The moderator set as a whole was statistically significant [QM(5) = 6.26, *p* = 0.0044] and accounted for a substantial proportion of the between-study heterogeneity (*R^2^* ≈ 69.8%). Exercise intensity emerged as a strong negative predictor of effect sizes (*β* = −1.74, SE = 0.38, *t*(12) = −4.58, *p* = 0.0006, 95% CI [−2.57, −0.91]), indicating that higher exercise intensities were associated with smaller effects of music during acute exercise. Mean participant age was also negatively associated with effect sizes (*β* = −0.05, SE = 0.02, t(12) = −2.56, *p* = 0.0248, 95% CI [−0.09, −0.01]), whereas sample size showed a trend towards smaller effects in larger studies (β = −0.03, SE = 0.01, t(12) = −2.05, *p* = 0.0625, 95% CI [−0.05, 0.00]). Music tempo and music selection were not statistically significant predictors (*ps* > 0.15). Despite the variance explained by the moderator set, substantial residual heterogeneity remained (QE(12) = 71.39, *p* < 0.001; *τ*^2^ = 0.37; *I*^2^ = 93.56%). Given the small number of studies and the presence of multiple effect sizes per study, these findings should be interpreted cautiously.

**Table 5:** Meta-regression of effect sizes on study-level moderators (*k* = 18).

Predictor	Estimate	Standard error	*t*	df	*p*	Lower	Upper
						95% CI	
Intercept	6.32	2.35	2.69	12	0.0196	1.20	11.443
Study size	−0.03	0.01	−2.05	12	0.0625	−0.05	0.00
Participant age	−0.05	0.02	−2.56	12	0.0248	−0.09	−0.01
Music tempo	0.58	0.46	1.26	12	0.2304	−0.42	1.59
Music selection	−0.74	0.49	−1.52	12	0.1536	−1.80	0.32
Exercise intensity	−1.74	0.38	−4.58	12	0.0006	−2.57	−0.91

Coefficients estimated using a mixed-effects model with REML and Knapp–Hartung adjustment.

#### 3.7.2 Exercise intensity: exploratory categorical model

Because the multivariable model was unstable and exercised limited power, we examined exercise intensity more directly by fitting an exploratory model treating intensity as a three-level categorical moderator (*k* = 18). The omnibus moderator test did not reach conventional significance (*F*(2,15) = 2.77, *p* = 0.094), although the pattern of estimated effects followed a descending gradient from low, moderate, high intensity. Substantial residual heterogeneity remained (*τ*^2^ = 1.95; *I*^2^ = 98.89%).

Predicted effects from this model are presented in Table 6. Music showed its largest estimated effect at low intensities, with diminished effects at moderate and high intensities. However, the prediction intervals were wide, indicating considerable uncertainty around all pooled estimates.

**Table 6:** Predicted effect sizes by exercise intensity (categorical model).

Exercise intensity	*g* (predicted)	Standard error	95% CI	95% PI
Low exercise intensity	3.99	1.08	[1.10, 5.69]	[−0.36, 7.15]
Moderate exercise intensity	0.99	0.41	[0.11, 1.87]	[−2.11, 4.09]
High exercise intensity	0.28	0.85	[−1.53, 2.08]	[−3.20, 3.76]

Predictions derived from a mixed-effects model with exercise intensity as a three-level factor.

## 4 Discussion

This review examines the intersection of music and acute exercise by examining their potential interaction on executive functions and affective responses. Narrative findings indicate mixed results, with reported effects across executive function domains (e.g., inhibitory control and working memory) appearing inconsistent and context dependent. Meta-analyses indicate no significant effects on attention allocation, inhibitory control and core affect outcomes. Subgroup comparisons suggested a descriptive pattern in which effects appeared larger at low exercise intensities and attenuated as intensity increased. Although substantial heterogeneity persisted and the models were based on a small number of heterogeneous studies, the moderator analyses suggested that part of the between-study variance may be attributable to exercise intensity and participant age, and these signals should be viewed as tentative.

Due to the aforementioned mixed results and heterogeneity across studies, the findings of this review are inconclusive. While prediction intervals reveal substantial variability in potential true effect sizes (ranging from negative to large positive effects) this variability presents an uncertain, potentially context-dependent relationship between music, acute exercise, executive functions and affective responses. The heterogeneity may further indicate the complexity of executive function interactions (e.g., unity and diversity, Miyake et al., 2000; Mackie et al., 2013), as well as the potential for individualised responses, warranting further investigation.

### 4.1 Potential mechanisms underlying music-exercise effects on executive function and affective outcomes

Before discussing the findings with broader associated theoretical models (e.g., DMT and the effort hypothesis), it is important to acknowledge that this review exclusively examined executive functions (e.g., attention allocation, inhibitory control, and cognitive flexibility) subserved by frontal-lobe-dependent processes (Smiley-Oyen et al., 2008; West, 2000; West, 1996). While these are core domains of executive functioning, this constrained scope of included functions precludes broader inferences regarding the full spectrum of executive functioning (Diamond, 2013). As such, our interpretations are restricted to the specific executive components examined, and speculative. Given the small number of heterogeneous studies, any links to mechanistic models must remain cautious. Nonetheless, we discuss how the observed outcomes may correspond with the theoretical premises of DMT, and the effort hypothesis.

### 4.2 Dual-mode theory and the effort hypothesis

Although the moderator analyses provide only preliminary evidence that exercise intensity is related to effect sizes, the narrative synthesis and subgroup estimates are consistent with a pattern in which music shows larger effects at low intensities and attenuated effects as intensity increases. Speculatively, the intensity-dependent influence may arise from interacting physiological and psychological mechanisms that vary as a function of exercise intensity (Ekkekakis, 2003; Karageorghis and Priest, 2012). At low-to-moderate exercise intensities, the cognitive demands of exercise are lower, leaving additional cognitive resources available for processing external stimuli such as music (Terry et al., 2020). This finds alignment with DMT’s hypothesis that lower exercise intensities allow for consistent cognitive-affective interaction within exercise, due to tolerable physiological and cognitive demands (Ekkekakis, 2003). Music and acute exercise were associated with elevated dissociative attention following low-to-moderate intensity tasks (as well as recovery periods) (Bigliassi et al., 2018, 2019; Feiss et al., 2021; Marques et al., 2022), and those effects diminished under high-intensity or static conditions, where associative attention and interoceptive salience likely dominated (Feiss et al., 2021; Marques et al., 2022). Mixed findings regarding inhibitory control (Chang et al., 2013; Chen et al., 2021; Jones et al., 2021; Suwabe et al., 2021) and working memory (Chen et al., 2021; Vaart, 2022) may also indicate dependency on exercise intensity due to interoceptive cues becoming more salient at higher intensities, which might have impaired cognitive performance.

An interesting finding to emerge from the meta-analysis data were the null results for core affect and subgroup analysis of affective valence, potentially attributable to the high interoceptive demands of exercise (e.g., Marques et al., 2022), mismatches in music tempo (e.g., Chen et al., 2021) or music selection (e.g., Tanaka et al., 2018), as well as variability in participant preferences and measurement methods across the included studies. Importantly, this pattern supports the view that affective valence and arousal are orthogonal dimensions (Russell et al., 1989). Specifically, the null affective arousal may paradoxically be more conducive to executive function engagement, whereas elevated affective valence may be optimal for improving exercise experiences. One potential explanation is that participants in these studies were already at or near optimal arousal levels (e.g., Tanaka et al., 2018; Bigliassi et al., 2018, 2019). This interpretation aligns with Easterbrook’s (1959) cue utilisation hypothesis and the Yerkes-Dodson Law (e.g., Yerkes and Dodson, 1908), which together posit that moderate levels of arousal support broader cognitive performance, whereas heightened arousal narrows and restricts the deployment of cognitive resources (Easterbrook, 1959; Yerkes and Dodson, 1908). Conceivably, the studies reporting improved performance on the executive function measures at these lower arousal levels [i.e., null affective arousal (Tanaka et al., 2018)] may indicate a supportive cognitive-affective state [e.g., facilitating improved executive function performance (Eysenck, 1982)], where the music-exercise protocol avoided surpassing an arousal threshold, thus diverting attention allocation and improving inhibition. Taken together, these findings provide conceptual insight into how the cognitive and affective domains of music-exercise may be constrained by intensity-dependent physiological and interoceptive demands that surpass the zone of response variability (Ekkekakis, 2003).

Another plausible interpretation is grounded in the effort hypothesis (Audiffren and André, 2019). Lower exercise intensities may have provided conditions under which executive functions were readily accessed (typically associated with deliberate, goal-directed Type 2 processing, e.g., Furley et al., 2015; Furley and Wood, 2016). Under these conditions, physiological stress is minimised, allowing additional cognitive resources to be allocated toward effortful processes (e.g., the inhibitory control to override the impulse to disengage due to fatigue) (Audiffren and André, 2019; Furley et al., 2015; Furley and Wood, 2016).

In addition, the narrative synthesis points to the contextual nature of these observed patterns. For instance, Chang et al. (2013) found that high-intensity cycling improved Stroop performance, but concurrent exposure to high-decibel music did not enhance inhibitory control beyond exercise alone and was linked to reduced prefrontal oxygenation. This suggests that not all music manipulations are beneficial; volume and stimulus characteristics can negate or obscure any potential cognitive advantages. Similarly, Jones et al. (2021) observed no significant effects using researcher-selected music after high-intensity interval training. These divergent findings point to a sensitivity of music-exercise interactions with contextual factors, such as specific music-exercise protocols, auditory characteristics, and task design. Regarding affective outcomes, mismatches between music tempo and participant musical preferences (e.g., Tanaka et al., 2018) or the use of high-intensity protocols (e.g., Feiss et al., 2021; Marques et al., 2022) appeared to diminish music’s influence on cognitive outcomes.

### 4.3 Factors contributing to study heterogeneity

Variability in music (e.g., the use of self-selected and/or researcher-selected music) and exercise protocols emerged as a significant source of heterogeneity (see, e.g., Supplementary Tables S1, S2). Self-selected music often aligns more closely with individual preferences and physiological rhythms, eliciting stronger affective and cognitive responses, as demonstrated by Karageorghis et al. (2021b), where participant-selected slow-tempo music elevated affective responses by reducing mental demand during tasks. In contrast, mismatched protocols, such as slow-tempo music during high-intensity exercise, diminished both cognitive and affective outcomes (Chen et al., 2021; Tanaka et al., 2018). Differences among participants not accounted for in the meta-regression analysis, such as fitness level and cultural background may have influenced the outcomes of the music-exercise combination (Basso et al., 2022; Kramer and Colcombe, 2018; Smiley-Oyen et al., 2008). For instance, individuals at higher fitness levels may be better able to sustain these cognitive benefits at higher exercise intensities by mitigating the effect of reaching VT (e.g., the point at which breathing becomes significantly more labored during exercise) (Basso et al., 2022). These differences influence how individuals experience music and exercise simultaneously, as affective responses, auditory-motor coupling, and tolerance for exercise intensity may influence which cognitive resources can be directed toward such tasks (Hutchinson et al., 2018; Hutchinson and Karageorghis, 2013; Terry et al., 2020).

In addition, how the studies operationalised discrete executive function components, which were often measured by isolated tasks that assume non-overlapping processes may present a methodological limitation. As noted by Posner and Presti (1987), Miyake et al. (2000), and Mackie et al. (2013) this presents challenges, as there is much evidence to suggest overlap in these functions, raising concerns about the measurement of treating them as wholly distinct constructs. Similarly, widely different measurement tools used across the studies contributed to heterogeneity. Tasks such as the Stroop test, while broadly used, overlap in assessing inhibitory control and attention allocation (Hagyard et al., 2021), introducing potential confounds and masking domain-specific effects. Latent constructs within these tools, such as mood and arousal influences, may have further magnified variability (Imbir et al., 2017). To this point, the reliability and validity of the measures used in these studies are generally well-supported, but their appropriateness for specific exercise contexts warrants scrutiny. Contextual factors such as participant fatigue or environmental conditions can compromise data reliability if the scales were not explicitly designed for such scenarios (Ekkekakis and Petruzzello, 2000). The critical issue is whether researchers selected and adequately justified instruments that were optimal for the contexts under investigation. Many studies relied on previously validated scales without sufficiently addressing their relevance to specific populations or contexts (Jones et al., 2021; Marques et al., 2022). This overreliance risks misalignment between scale design and study context. Additionally, administration methods, such as self-reports pre- post- exercise, may introduce common method biases (e.g., variance transfer) inflating variance and heterogeneity in the meta-analytic findings.

Finally, our categorisation into three meta-analytic clusters (attention allocation, inhibitory control, and core affect) may have oversimplified the analysis of the constructs measured across studies, given the specific design choices underlying these constructs. This misalignment may have contributed to the high heterogeneity observed in the meta-analyses.

### 4.4 Limitations and implications

The applicability of the present review is limited to recreationally active populations. Accordingly, further examination of music and acute exercise with at-risk populations [e.g., cardiovascular disease (Alter et al., 2015)] and those that are insufficiently active, appears warranted. The exploratory focus and high heterogeneity across the included studies and meta-analyses precludes the ability to draw definitive causal conclusions. One retained study (Chen et al., 2021) used a mismatched music condition (e.g., slower or faster tempo) in place of a true no-music control. While this was initially accepted as a proxy comparator, we acknowledge that mismatched music may elicit its own unique cognitive-affective responses and therefore does not constitute a neutral baseline. This deviation from the intended comparator introduces interpretive limitations and should be considered when evaluating the consistency of findings across studies. Similarly, variability in study methodologies, including differences in exercise intensity, music protocols, outcome measurements, and participant characteristics, likely contributed to the heterogeneous results. Although extensive efforts were made to minimise publication bias through comprehensive database searches, the exclusion of conference abstracts and non-English language studies introduces the potential for language and selection biases.

Only 10 studies were included in the review, with nine eligible for meta-analysis, limiting its scope and reducing the breadth of the quantitative synthesis given the relatively small number of studies. A high risk of bias study was also retained for synthesis (e.g., Jones et al., 2021) due to the exploratory scope of the review. Synthesizing data from a small number of studies may constrain the precision of pooled estimates, reducing the reliability of moderator analyses (Valentine et al., 2010; Gurevitch et al., 2018). This extends to the several outcome domains explored in the present study, where fewer than 10 effect sizes were available. As the number of studies was small, we treated multiple effect sizes from the same study as independent in the meta-regression; this likely underestimates standard errors and reinforces the need to interpret moderation findings cautiously. In addition, despite large effect sizes, the wide confidence intervals and prediction intervals highlight substantial variability and uncertainty in the findings, reducing their statistical robustness.

The present data may indicate some utility in the adoption of low-to-moderate arousal music to match a low or moderate-intensity acute exercise protocol and could provide a novel avenue to examine the interaction between music’s influence on recuperation from exercise and executive functions. Passive recovery is intrinsic to many high-intensity exercise protocols, and further research is warranted to examine potential differences in optimal music-exercise approaches for enhancing recovery outcomes (Danso et al., 2025; Karageorghis et al., 2021a).

## 5 Conclusion

The reviewed evidence examining whether music contributes to changes in executive functions during acute exercise is inconclusive. While findings varied across domains, attention allocation and inhibitory control were the executive functions with the greatest number of analysable studies. Across both cognitive and affective domains, the available data are descriptively consistent with a pattern in which effects appear less pronounced at higher exercise intensities, but moderator tests were underpowered and non-significant. Any potential intensity-related attenuation of music effects should therefore be interpreted cautiously. Cumulatively, the findings of this review are to be interpreted cautiously, due to high heterogeneity and variability in study designs resulting in inconsistent findings across executive functions. Future research may prioritise high-quality RCTs with mediation analyses to explore the explicit influence of specific musical stimuli and acute exercise protocols on executive functions and affective responses.

## Data Availability

The original contributions presented in the study are included in the article/Supplementary material, further inquiries can be directed to the corresponding author.
