# Supplementary material for: Does music support executive functions and affective responses during acute exercise? A systematic review and meta-analysis
Source: Front Psychol. 2026 Jan 8;16:1714707. doi: 10.3389/fpsyg.2025.1714707 (PMC12823824; doi:10.3389/fpsyg.2025.1714707)
Supplement: Supplementary file 1 [file Supplementary_file_1.docx]

**Appendix**

**Appendix A. Summary of operationalisation of main outcomes of interest in the review**

The main outcomes of interest in the review are operationalised as follows:

- **Attention allocation** in physical exercise involves directing focus towards internal sensations (association) and/or external stimuli (dissociation) (Tammen, 1996). Evidence suggests that individuals allocate attention to both internal and external cues simultaneously, with the degree of focus on each type of stimuli being context-dependent (Tenenbaum & Connolly, 2008).
- **Cognitive flexibility** in physical exercise refers to the dynamic interplay of attentional control and strategy adjustment, enabling individuals to effectively adapt to the evolving physical and mental demands of exercise. This involves the ability to shift focus between internal and external stimuli, modify exercise strategies based on feedback and changing conditions, and regulate cognitive processes (Diamond, 2013; Braem & Egner, 2018).
- **Core affect** is the fundamental experience of feeling, encompassing both emotions and moods (Hardy & Rejeski, 1989). It is characterised by two core dimensions: valence (pleasure-displeasure) and activation (arousal-sleepiness). Affect arises from a complex interplay of physiological processes, cognitive appraisals, and situational influences, and its expression can range from basic, reflexive responses to complex, nuanced emotions.
- **Inhibitory control**, crucial for suppressing impulsive actions (such as abrupt changes in intensity or technique) and resisting distractions from both internal (e.g., negative thoughts) and external sources (e.g., environmental factors), is essential for regulating attention and making deliberate decisions during physical exercise (Diamond, 2013; Tiego et al., 2018).
- **Task switching**, a component of cognitive flexibility, involves the coordinated interplay of attentional functions, including interference suppression, to enable shifts between different cognitive and motor skills (Diamond, 2013). The efficiency and speed of task switching are influenced by factors like task complexity, individual differences, and practice, and play a crucial role in adapting to the dynamic demands of physical exercise.
- **Working memory**, a system that actively holds, manipulates, and processes information, is essential for retaining and applying critical information during physical exercise, including instructions, goals, pacing, and technique (Baddeley, 1992; Diamond, 2013). It enables individuals to monitor physiological feedback, perform mental calculations, plan and execute movement sequences, and adapt to dynamic exercise demands. Working memory shares neural mechanisms with attention, relying on interconnected brain regions such as the prefrontal and parietal cortex to support executive functions during physical exercise (Baddeley, 1992; Diamond, 2013).

## **Appendix B. Summary of findings of Randomised Controlled Trials**

Two RCTs (Chen et al., 2021; Jones et al., 2021) investigated the effect of music during acute exercise on various cognitive processes and affective outcomes, examining outcomes in inhibitory control , working memory and core affect. Across the trials, music used during exercise was consistently associated with improvements in inhibitory control and working memory (measured post-exercise) compared to exercise without music. (Chen et al., 2021) measured inhibitory control immediately after a 20-minute, moderate-intensity aerobic exercise session. (Jones et al., 2021) measured inhibitory control using the Stroop test within 30 seconds of exercise cessation, with all participants finishing within three minutes (see e.g., Table S1 for a summary of the studies).

**Table S1. Summary table of included studies that were RCTs.**

| **Study** | **Music Protocol** | **Exercise Protocol** | **Identified Executive Function and Affective Outcomes** | **Cognitive or Affective Measurement** |
| --- | --- | --- | --- | --- |
| Chen et al. (2021) | Participants listened to music without lyrics at tempos of 60-65 BPM (slow), 120-140 BPM (moderate), or 155-165 BPM (fast) during exercise. The music tempo was either matched or mismatched with heart rate. | Participants completed 20 minutes of moderate-intensity cycling at 60-70% of their maximum heart rate (HRmax). Three groups were assigned based on whether the music tempo was slower, matched, or faster than their HR. | Inhibitory control  Working memory  Core Affect (Valence) | Stroop task  n-back task (1-back and 2-back), and more-odd shifting task  Check List (CMACL) |
| Jones et al. (2021) | Participants listened to either no music, classical (sedative), or rock (stimulative) music during both control and high-intensity exercise sessions. Music exposure was randomised. | Participants performed a 12-minute high-intensity interval training (HIIT) session. Heart rates were monitored, and participants completed the Stroop test immediately after exercise. | Inhibitory Control | Stroop Task |

## **Appendix C. Summary of findings of experimental studies**

Eight studies used a variety of experimental designs to examine the effects of music listening during acute exercise on executive functions and affective outcomes, reporting outcomes across attention allocation (Bigliassi et al., 2018, 2019; Feiss et al., 2021; Marques et al., 2022), core affect (Bigliassi et al., 2018, 2019; Feiss et al., 2021; Suwabe et al., 2021; Marques et al., 2022), inhibitory control (Chang et al., 2013; Suwabe et al., 2021), and working memory (Vaart, 2022) (see e.g., Table S2 for a summary of the studies).

**Table S2. Summary table of included studies with other experimental designs than RCTs.**

| **Study** | **Music Protocol** | **Exercise Protocol** | **Identified Executive Function and Affective Outcomes** | **Cognitive or Affective Measurement** |
| --- | --- | --- | --- | --- |
| Bigliassi et al. (2019) | Participants listened to the song “I Heard It Through the Grapevine” (119 BPM) by Creedence Clearwater Revival during a 10-minute isometric handgrip exercise session. The music was delivered through MRI-compatible earphones at a sound intensity of ~75 dBA. | Participants performed 30 trials of a 10-second isometric handgrip exercise at 30% of their maximal voluntary contraction (MVC), followed by 10 seconds of rest. The exercise was performed while inside an MRI scanner to measure brain activity during the task. | Attention Allocation  Core Affect (Arousal) | Attentional Scale (Tammen’s scale)  Felt Arousal Scale |
| Bigliassi et al. (2018) | Participants listened to the song “Happy” (160 BPM) by Pharrell Williams during a 6-minute outdoor walking session on a 400-meter running track at a self-paced speed. | Participants walking 400 meters at a self-paced speed on a standard outdoor running track. Each participant completed this walking task under three different conditions: music, podcast, and control (no auditory stimulus). The auditory stimuli were randomised and counterbalanced across participants. | Attention Allocation  Core Affect (Arousal) | Attentional Scale (Tammen’s scale)  Felt Arousal Scale |
| Chang et al. (2013) | Participants listened to researcher-chosen, high-decibel music at 100 dB, considered high-intensity sound (HIS), during exercise sessions. | Participants cycled on an ergometer at 70-75% of VO_2_ max (a level that constitutes high-intensity exercise). | Inhibitory Control | Stroop Task |
| Feiss et al. (2021) | Participants listened to either fast-tempo music (120 BPM) or slow-tempo music (90 BPM), with tracks selected from the Billboard Hot 100 Chart. The music was played throughout the duration of the exercise at a constant volume of 75 dBA using external speakers. The fast and slow-tempo tracks were edited to maintain consistent tempos, ensuring the differentiation between music conditions. A no-music control condition was also used for comparison. | Participants performed two isometric strength exercises: a wall-sit and a plank-hold. In the wall-sit, participants maintained a seated position with their back against the wall and knees at a 90-degree angle, holding the position until voluntary exhaustion. For the plank-hold, participants held a prone plank position, supported by their elbows and feet, until they could no longer maintain proper form. Both exercises were completed in a baseline (no-music) trial, followed by an experimental trial with either no music, fast-tempo music, or slow-tempo music. | Attention Allocation  Core Affect (Arousal & Valence) | Attentional Scale (Tammen’s scale)  Affect Grid |
| Marques et al. (2022) | Participants completed three conditions: self-selected music (high-tempo favorite tracks), randomly selected music (a "Sport" playlist), and no music. Music (140-160 BPM) played during the 5-minute warm-up, exercise bouts, and recovery periods via headphones, with volume standardised at 75%. Music's motivational properties were evaluated post-session. | The sprint interval training (SIT) involved 8 × 15-second all-out cycling bouts at 9% body mass resistance, with 120 seconds of passive recovery between bouts. Performance metrics (peak power, mean power, fatigue index, and total work) were recorded, and no verbal encouragement was provided. Sessions were spaced 72 hours apart for recovery. | Attention Allocation  Core Affect (Valence) | Attentional Scale (Tammen’s scale)  Feeling Scale |
| Suwabe et al. (2021) | Participants listened to their favourite music or metronome beeps during 10 minutes of moderate-intensity pedalling exercise. The music had a tempo of 120 beats per minute, synchronised with the pedalling. | Participants performed 10 minutes of moderate-intensity pedalling exercise (50% of their peak oxygen uptake) on a recumbent cycle ergometer at 60 revolutions per minute. | Inhibitory Control  Core Affect (Valence) | CWST  Two-Dimensional Mood Scale (TDMS) |
| Tanaka et al. (2018) | Participants listened to self-selected music through headphones during the exercise. Songs were Japanese pop music with a tempo below 120 BPM. The volume was set to 80 dB. | Participants performed 30 minutes of moderate-intensity cycling at 60% VO_2_ peak. They maintained a cadence of 60 revolutions per minute. | Inhibitory Control  Core Affect (Arousal) | Stroop Task  Felt Arousal Scale |
| Vaart (2022) | Participants listened to a 30-minute classical music playlist (120-140 BPM) with noise-cancelling headphones at a consistent volume during the session. | Participants exercised on a recumbent cycle ergometer for 30 minutes at a moderate intensity (55% of their heart rate reserve), including a 5-minute warm-up and cool-down. | Inhibitory Control  Working Memory | Stroop Task  Reverse Corsi Block Task |

## **Appendix D. Publication bias: sensitivity analysis**

A sensitivity analysis was conducted to assess publication bias by relating standard errors to effect size estimates across the outcomes of interest. Following the recommendations of (Sterne & Egger, 2001; Sterne & Harbord, 2004), a series of funnel plots of per-outcome standard error by standard difference in group means was produced and assessed for evidence of asymmetry. Forest plots summarised the effect size data, while funnel plots were used to explore potential publication bias.

Egger’s test (Egger et al., 1997) indicated significant asymmetry for attention allocation (*z* = 2.953, *p* = .003), significant asymmetry for inhibitory control (*z* = 4.796, *p* < .001), and significant asymmetry for core affect outcomes (*z* = 3.275, *p* = .001) (Figure S1). Because of potential publication bias, the summary effect sizes for attention allocation, inhibitory control and core affect outcomes may thus be slightly inflated.


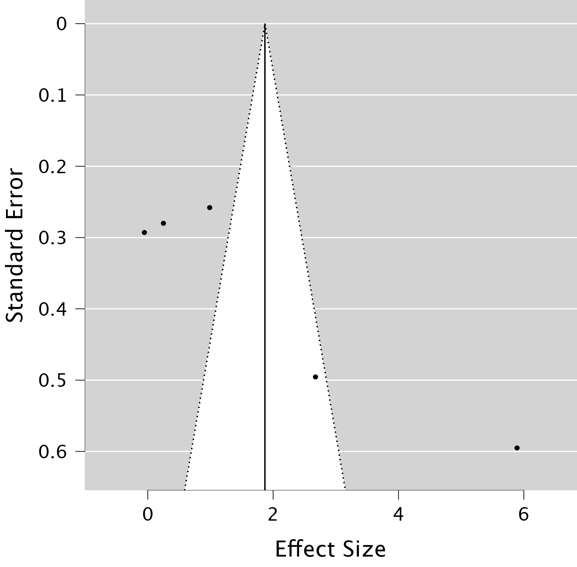

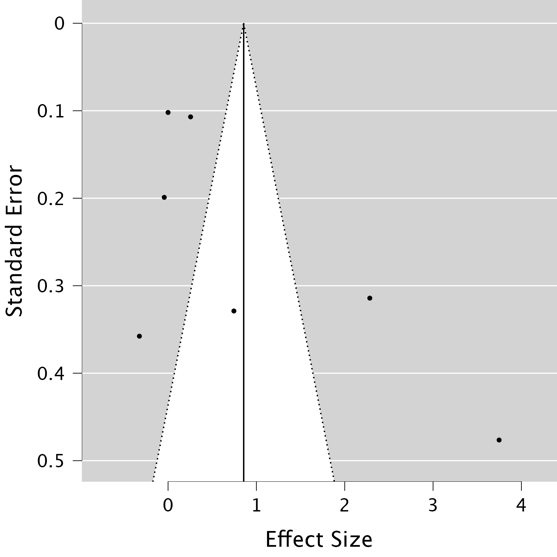

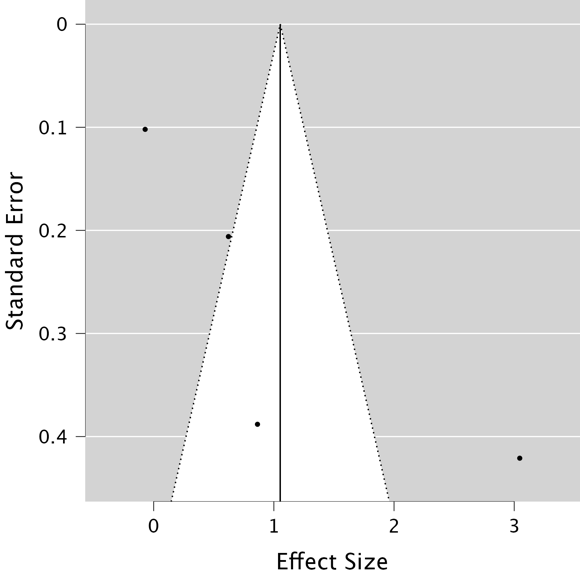


**Attention Allocation**

**Inhibitory Control**

**Core Affect**

**Figure S1. Funnel plots for attention allocation, inhibitory control and core affect outcomes.**

**Appendix References**

Baddeley A. 1992. Working Memory. *Science* 255:556–559. DOI: 10.1126/science.1736359.

Bigliassi M, Karageorghis CI, Bishop DT, Nowicky AV, Wright MJ. 2018. Cerebral effects of music during isometric exercise: An fMRI study. *International Journal of Psychophysiology* 133:131–139. DOI: 10.1016/j.ijpsycho.2018.07.475.

Bigliassi M, Karageorghis CI, Hoy GK, Layne GS. 2019. The Way You Make Me Feel: Psychological and cerebral responses to music during real-life physical activity. *Psychology of Sport and Exercise* 41:211–217. DOI: 10.1016/j.psychsport.2018.01.010.

Braem S, Egner T. 2018. Getting a Grip on Cognitive Flexibility. *Current Directions in Psychological Science* 27:470–476. DOI: 10.1177/0963721418787475.

Chang H, Kim K, Jung Y-J, Ahn N-R, So W-Y, Kato M. 2013. Effects of blood flow to the prefrontal cortex on high-intensity exercise combined with high-decibel music. *Journal of Exercise Nutrition and Biochemistry* 17:115–122. DOI: 10.5717/jenb.2013.17.4.115.

Chen J, Su R, Lv Z, Xiao J, Zhao Y, Wang D, Jiang E. 2021. The effect of acute aerobic exercise with music on executive function: The major role of tempo matching. *Physical Activity and Health* 5:31–44. DOI: 10.5334/PAAH.75.

Diamond A. 2013. Executive Functions. *Annual Review of Psychology* 64:135–168. DOI: 10.1146/annurev-psych-113011-143750.

Egger M, Smith GD, Schneider M, Minder C. 1997. Bias in meta-analysis detected by a simple, graphical test. *bmj* 315:629–634.

Feiss R, Kostrna J, Scruggs JW, Pangelinan M, Tenenbaum G. 2021. Effects of music tempo on perceived exertion, attention, affect, heart rate, and performance during isometric strength exercise. *Journal of Sports Sciences* 39:161–169. DOI: 10.1080/02640414.2020.1809974.

Hardy CJ, Rejeski WJ. 1989. Not What, but How One Feels: The Measurement of Affect during Exercise. *Journal of Sport and Exercise Psychology* 11:304–317. DOI: 10.1123/jsep.11.3.304.

Jones D, Fariss J, Blaisdell R, Jimenez L, Morrison J. 2021. Selective attention is resistant to high intensity exercise and musical distraction. *Journal of Kinesiology & Wellness* 9:84–91. DOI: 10.56980/jkw.v9i.79.

Marques M, Staibano V, Franchini E. 2022. Effects of self-selected or randomly selected music on performance and psychological responses during a sprint interval training session. *Science & Sports* 37:139.e1-139.e10. DOI: 10.1016/j.scispo.2021.02.006.

Sterne JAC, Egger M. 2001. Funnel plots for detecting bias in meta-analysis. *Journal of Clinical Epidemiology* 54:1046–1055. DOI: 10.1016/S0895-4356(01)00377-8.

Sterne JAC, Harbord RM. 2004. Funnel Plots in Meta-analysis. *The Stata Journal: Promoting communications on statistics and Stata* 4:127–141. DOI: 10.1177/1536867X0400400204.

Suwabe K, Hyodo K, Fukuie T, Ochi G, Inagaki K, Sakairi Y, Soya H. 2021. Positive Mood while Exercising Influences Beneficial Effects of Exercise with Music on Prefrontal Executive Function: A Functional NIRS Study. *Neuroscience* 454:61–71. DOI: 10.1016/j.neuroscience.2020.06.007.

Tammen VV. 1996. Elite middle and long distance runners associative/dissociative coping. *Journal of Applied Sport Psychology* 8:1–8. DOI: 10.1080/10413209608406304.

Tanaka D, Tsukamoto H, Suga T, Takenaka S, Hamaoka T, Hashimoto T, Isaka T. 2018. Self-selected music-induced reduction of perceived exertion during moderate-intensity exercise does not interfere with post-exercise improvements in inhibitory control. *Physiology and Behavior* 194:170–176. DOI: 10.1016/j.physbeh.2018.05.030.

Tenenbaum G, Connolly CT. 2008. Attention allocation under varied workload and effort perception in rowers. *Psychology of Sport and Exercise* 9:704–717. DOI: 10.1016/j.psychsport.2007.09.002.

Tiego J, Testa R, Bellgrove MA, Pantelis C, Whittle S. 2018. A Hierarchical Model of Inhibitory Control. *Frontiers in Psychology* 9:1339. DOI: 10.3389/fpsyg.2018.01339.

Vaart JV. 2022. The Effects of Exercise Combined with Music on Executive Functioning in Healthy Young Adults. University of Waterloo.
